# LUCIND: profiling respiratory comorbidity in paediatric neurological diseases

**DOI:** 10.1183/23120541.00081-2026

**Published:** 2026-10-26

**Authors:** Daniel A.F. Bernard, Luca Salhöfer, Florian Stehling, Cathrin Dahl, Ulrike Schara-Schmidt, Anna Pauly, Margarete Olivier, Matthias Welsner, Eva-Maria Huessler, Irma Kollenda, Bernd Schweiger, Sebastian Zensen, Johannes Haubold, Marcel Opitz, on behalf of the LUCIND Working Group

**Affiliations:** 1 Pediatric Pulmonology and Sleep Medicine, University Hospital Essen, University of Duisburg-Essen, Essen, Germany; 2 Institute of Diagnostic and Interventional Radiology and Neuroradiology, University Hospital Essen, Essen, Germany; 3 Department of Paediatric Neurology, Centre for Neuromuscular Disorders in Children and Adolescents, University Hospital Essen, University of Duisburg-Essen, Essen, Germany; 4 Department of Pulmonary Medicine, University Hospital Essen-Ruhrlandklinik, Essen, Germany; 5 Institute for Medical Informatics, Biometry and Epidemiology, University Hospital Essen, Essen, Germany

## Abstract

**Background:**

Children and adolescents with neurodisability are at high risk of respiratory comorbidity and secondary pulmonary disease due to impaired airway clearance, thoracic restriction, dysphagia and microbial airway colonisation. Despite the clinical relevance of these complications, systematic imaging data on structural lung changes and their clinical correlates in this population remain scarce.

**Methods:**

LUCIND (Lung Comorbidity in Neurological Diseases) is a prospective, cross-sectional imaging study enrolling 150 children and adolescents with neurodisability without primary pulmonary disease. Participants are stratified into three risk-based subgroups (n=50 each): 1) neurodisability with tracheostomy, 2) neurodisability with dysphagia, and 3) neurodisability without dysphagia. The primary objective is to detect chronic structural lung abnormalities such as bronchiectasis, bronchial wall thickening and consolidations using ultralow-dose photon-counting computed tomography. This technique yields robust morphological data at radiation levels approaching those of conventional radiography. Due to ultra-short acquisition times, imaging is performed without sedation, ensuring high patient safety and feasibility in this vulnerable population. Secondary aims include the correlation of radiological findings with clinical features, such as swallowing function, respiratory infection burden, microbial airway colonisation, and therapeutic measures including airway clearance and antibiotic use. Imaging will be complemented by standardised clinical data, logopaedic assessment, and detailed caregiver questionnaires.

**Conclusion:**

LUCIND will provide a unique dataset to refine our understanding of chronic lung disease in children with neurodisability. Results may inform future risk stratification, early intervention strategies, and optimised clinical pathways, potentially improving both quality of life and long-term respiratory outcomes.

## Shareable abstract

**The LUCIND study uses ultralow-dose photon-counting CT to define the respiratory phenotype in children with neurodisability, providing robust lung imaging without sedation at radiation doses comparable to conventional radiography** https://bit.ly/4tOsDRy

## Introduction

Children and adolescents with neurodisability are at high risk of respiratory morbidity and mortality [1–6]. In these patients, respiratory diseases cause frequent hospital readmissions, significantly increasing disease burden and healthcare utilisation [2]. Life expectancy has increased significantly over recent decades, mainly due to advances in respiratory management, particularly the proactive introduction of home mechanical ventilation and mechanical insufflation–exsufflation [3, 5]. However, secondary chronic lung disease remains insufficiently defined [7]. Structural pulmonary abnormalities such as bronchiectasis, bronchial wall thickening, mucus plugging, and air trapping are increasingly recognised, but poorly characterised in this vulnerable group [8].

In children with neurodisability, pulmonary disease develops secondary to ineffective airway clearance. Impaired mucociliary clearance in this population is mainly driven by cough insufficiency in the context of thoracic restriction and expiratory muscle weakness [9], dysphagia due to bulbar dysfunction [10, 11], and tracheostomy-related limitation of secretion elimination [12]. Ineffective clearance promotes bacterial airway colonisation and chronic neutrophilic bronchitis [13]. In otherwise healthy children, this condition is known as protracted bacterial bronchitis (PBB) and is considered to be the precursor of non-cystic fibrosis bronchiectasis [14]. Bronchiectasis represents the irreversible end-stage of numerous chronic lung diseases. The pathological dilation of the bronchi is associated with retained, viscous secretions, creating a self-perpetuating cycle of infection, inflammation and airway destruction [15]. The structural and inflammatory airway changes in neurologically impaired children may span a heterogeneous spectrum of chronic suppurative lung disease, ranging from PBB to persistent atelectasis and irreversible bronchiectasis [16]. PBB remains underdiagnosed due to nonspecific symptoms and limited patient communication. In addition, progressive loss of vital capacity [17, 18], altered pulmonary compliance [19], and thoracic skeletal deformities with reduced regional ventilation [5] further contribute to impaired mucociliary clearance. Scoliosis-driven stenoses of the central airways may further impair the mobilisation of airway secretions [20].

Despite multiple factors causing secretion retention in children with neurodisability, systematic lung imaging data remain scarce. Computed tomography (CT) is the reference standard for imaging lung pathologies [21]. Conventional chest radiography lacks sufficient sensitivity and specificity for reliably detecting subtle findings, such as early bronchiectasis [22]. In cystic fibrosis, airway colonisation and infection are closely associated with structural CT abnormalities that correlate with the disease. However, comparable evidence for neurologically impaired children is almost entirely lacking. This knowledge gap is partly due to the exclusion of these patients from prior imaging cohorts, despite the critical role of respiratory morbidity. Our recent retrospective data [23] suggest that structural abnormalities, particularly lobar consolidations and bronchiectasis, are present in nearly all symptomatic children with neurodisability. Crucially, these findings were frequently missed on conventional chest radiographs, yet their detection *via* CT prompted immediate management changes in most cases.

Evidence from adult cystic fibrosis and non-cystic fibrosis bronchiectasis cohorts demonstrates that early detection and targeted treatment of airway structural changes can alter disease trajectory [8, 24]. Interventions such as rigorous implementation of airway clearance techniques and infection control reduce disease progression and, in early stages, may induce partial regression [8, 12, 25]. Better control of airway inflammation correlates with improved quality of life and fewer pulmonary exacerbations [8, 15, 26]. Nevertheless, current treatment recommendations for children with neurological disorders often rely on a presumed diagnosis of chronic non-cystic fibrosis bronchiectasis rather than confirmed radiological evidence [27].

In recent years, photon-counting detector CT (PCCT) technology has revolutionised imaging with significant dose reduction at competitive image quality or higher resolution without a relevant dose penalty [28]. Therefore, PCCT enables high-resolution depiction of fine bronchial structures at radiation doses as low as 0.2 mSv, which is comparable to the radiation dose of a two-view chest radiograph [29–31]. Because PCCT detects individual photons and eliminates electronic noise, radiation-dose levels can be reduced far beyond what is achievable with conventional detectors, without compromising the assessment of clinically relevant findings. This aligns optimally with the “as low as reasonably achievable” principle for paediatric thoracic imaging.

To address these diagnostic and therapeutic gaps, the LUCIND (Lung Comorbidity in Neurological Diseases) study aims to prospectively establish the overall prevalence of and systematically characterise structural lung abnormalities in children with neurodisability. By integrating ultralow-dose PCCT with clinical and functional parameters, and standardised caregiver questionnaires, the study seeks to define clinical and radiological phenotypes to identify potential markers for early therapeutic intervention.

To our knowledge, LUCIND is the first prospective study to investigate structural lung disease in this specific population using ultralow-dose PCCT, bridging the gap between clinical complexity and detectable structural lung pathology through dose-optimised morphological assessment.

## Methods

### Participants

Participants of the LUCIND study are children and adolescents affected by the umbrella term neurodisability, a concept prevalent in British and European paediatric care focusing on children with complex physical impairments of the brain and/or neuromuscular system [32]. This framework prioritises shared clinical needs over diagnostic labels, encompassing both central nervous system (CNS) disorders and neuromuscular diseases (NMD), including individuals without a specific diagnosis [32]. To characterise clinical severity, we operationalise the Children with Medical Complexity framework recognised by the American Academy of Pediatrics [33]. Inclusion required the simultaneous fulfilment of four mandatory domains: chronic conditions with high morbidity, severe functional limitations, high healthcare utilisation, and significant family-identified needs. While many participants align with the criteria for severe neurological impairment [34], we acknowledge that this construct primarily reflects CNS-related deficits and may not fully capture the clinical reality of the NMD subgroup, where cognitive functions are often preserved. Severe motor impairment predominantly characterised by Gross Motor Function Classification System levels IV and V in the CNS group, and nonambulatory status in the NMD group serves as the primary driver for respiratory fragility [5, 35]. In alignment with the World Health Organization International Classification of Functioning, Disability and Health domain of body structures and functions, participants demonstrate profound impairments in airway clearance, bulbar function, and thoracic excursion, necessitating dependence on technology, and intensive multidisciplinary care [36].

### Identification and recruitment

We aim to include 150 children aged 5–18 years with neurodisability, encompassing a wide spectrum of neuromuscular and CNS disorders. Central neurological conditions include congenital malformation syndromes, cerebral palsy and epileptic encephalopathies. Neuromuscular conditions comprise motor neuron diseases (*e.g.* spinal muscular atrophy), congenital myopathies, and muscular dystrophies. All participants are suspected to have secondary structural lung disease due to sustained or progressive motor and/or bulbar dysfunction. Inclusion requires clinical indicators of lower airway involvement, such as chronic cough, recurrent respiratory tract infections, or other current or historical signs suggestive of chronic pulmonary disease. All participants can be assigned to one of three clinically relevant subgroups: 1) children with neurodisability and tracheostomy, focusing on the impact of chronic bacterial colonisation and bypass of upper airway defences; 2) children with neurodisability and dysphagia, focusing on lung injury secondary to (micro-)aspiration and chronic oropharyngeal dysfunction; 3) children with neurodisability without symptoms of dysphagia, focusing on isolated impairment of mucociliary clearance due to cough insufficiency and thoracic restriction.

This stratification allows for a comparative analysis of distinct mechanistic pathways leading to structural lung disease. Dysphagia-associated chronic aspiration is linked to specific microbial shifts and increased pathogen detection, further contributing to the pathogenesis of aspiration-related lung disease [37]. We exclude individuals with known primary pulmonary disease (*e.g.* cystic fibrosis, primary ciliary dyskinesia, or interstitial lung disease). Further exclusion criteria include acute severe illness precluding participation, pregnancy, increased individual susceptibility to ionising radiation and absence of informed consent. All imaging is performed during clinically stable intervals, defined as no acute respiratory exacerbations and no acute antibiotic therapy for ≥4 weeks prior to the scan. Participant flow is illustrated in figure 1.

**FIGURE 1:**
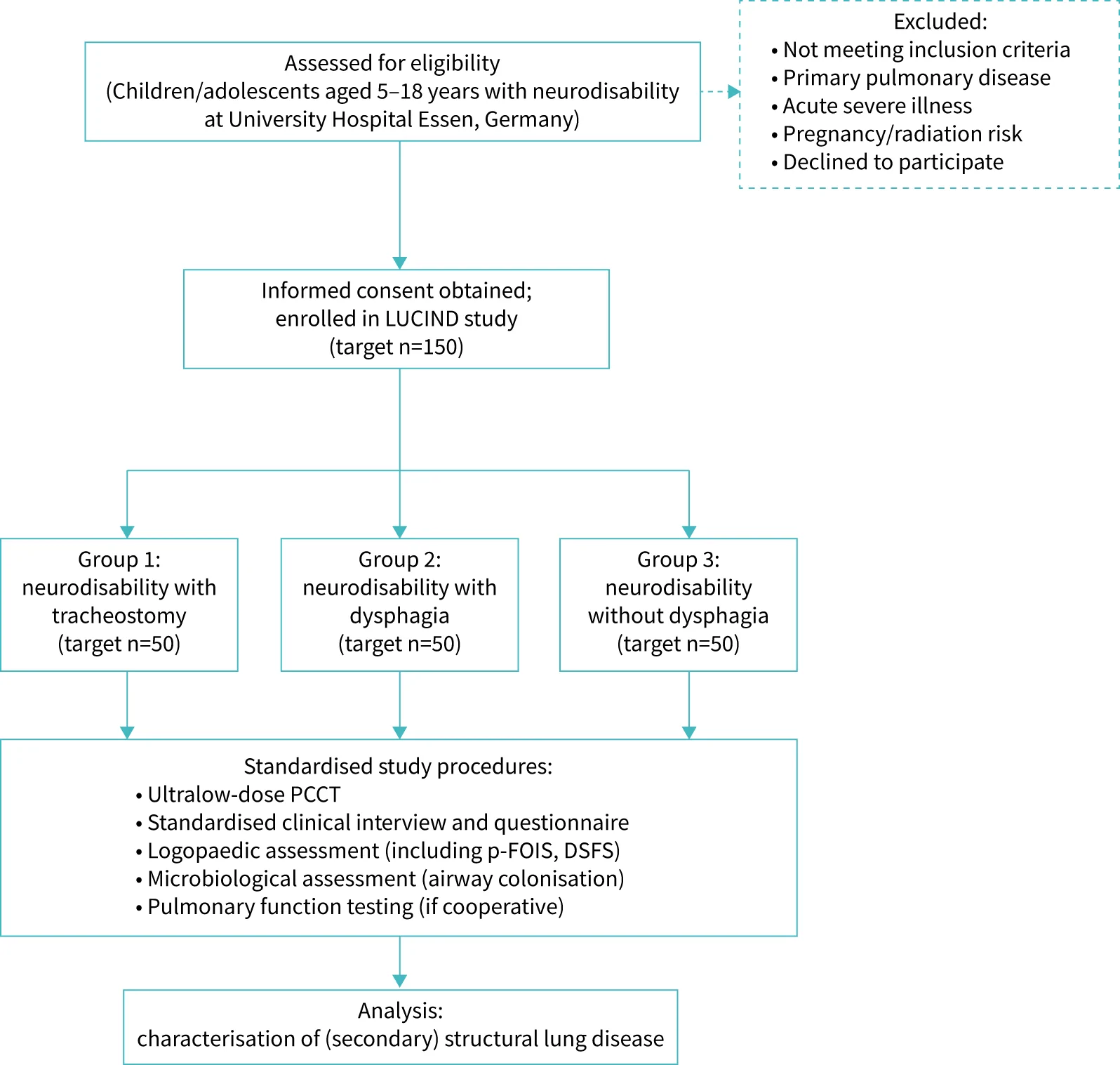
Study design, participant flow, and stratification process of the LUCIND (Lung Comorbidity in Neurological Diseases) study. Eligible children (aged 5–18 years) with neurodisability are screened and enrolled into three clinical subgroups based on their risk profile (tracheostomy, dysphagia or no dysphagia). All participants undergo a standardised assessment protocol including ultralow-dose photon-counting computed tomography (PCCT), clinical questionnaires, logopaedic evaluation, and microbiological airway sampling. p-FOIS: paediatric Functional Oral Intake Scale; DSFS: Drooling Severity and Frequency Scale.

### Data collection

#### Clinical data

A structured clinical assessment establishes baseline status across the domains detailed in table 1. We utilise a dual approach: first, a standardised semi-structured interview conducted by trained personnel to minimise recall bias. This interview involves the patient or primary caregiver, depending on communicative ability. Second, clinical records are reviewed for objective data on neurological diagnosis, disease severity, microbiology and comorbidities. All data are subsequently correlated with imaging findings to identify associations between patient characteristics and structural lung disease.

**TABLE 1:** Overview of variables and outcome measures collected in the LUCIND (Lung Comorbidity in Neurological Diseases) study

	Assessment method	Description/remarks
**Demographics and anthropometry**		
Age, sex	Clinical records	Used for stratification and adjustment
Weight, height, BMI	Clinical records/measurement	Basic anthropometric characterisation
Vaccination status	Questionnaire/vaccination card	Adherence to STIKO recommendations
**Neurological and functional status**		
Underlying diagnosis	Clinical records	Categorised: neuromuscular *versus* CNS origin
Mobility/motor function	Questionnaire	Walking distance, standing, sitting or bedridden
Daily life impairment	Questionnaire	Impact of respiratory situation on daily activities
**Respiratory history**		
Infection burden	Questionnaire/clinical records	Frequency of RTIs and antibiotic use (past 12 months)
Hospitalisation history	Records/questionnaire	Ward admissions and ICU stays due to respiratory events
Respiratory symptoms	Questionnaire	Frequency of dyspnoea/breathlessness
**Respiratory support**		
Ventilation details	Records/questionnaire	Invasive/NIV, continuous *versus* nocturnal, humidification, indication, time since initiation
Sleep study data	Clinical records	Nocturnal hypoventilation, when available
Airway clearance	Questionnaire	Cough assist (including oscillation), IPPB, VRP
Secretion management	Questionnaire	Spontaneous cough ability, suctioning frequency
Sputum characteristics	Questionnaire	Consistency (viscous/fluid), colour (purulent/clear)
**Medication**		
Respiratory medication	Records/questionnaire	Inhalation therapy (bronchodilators, steroids), nebuliser
Inhaled antibiotics	Records/questionnaire	Cycling or long-term therapy (*e.g.* tobramycin, colistin)
Immunomodulation	Records/questionnaire	Azithromycin maintenance therapy
Saliva control	Records/questionnaire	Glycopyrronium bromide, botulinum toxin A injections
Anti-reflux medication	Questionnaire	Proton pump inhibitors (*e.g.* omeprazole)
**Bulbar and GI function**		
Swallowing safety	p-FOIS scale	Paediatric Functional Oral Intake Scale
Drooling severity	DSFS scale	Drooling Severity and Frequency Scale
Reflux and vomiting	Questionnaire	Frequency of reflux symptoms and vomiting events
Feeding modality	Records/questionnaire	Oral intake, nasogastric tube, PEG/PEJ
**Environmental and social context**		
Household risk factors	Questionnaire	Tobacco smoke exposure, siblings aged <6 years
Care situation	Questionnaire	Nursing service involvement, daycare/school attendance
Therapy frequency	Questionnaire	Physiotherapy and logopaedics frequency
**Tracheostomy details (if applicable)**		
Cannula characteristics	Records/questionnaire	Age at placement, cuff time, speaking valve use
Complications	Questionnaire	Frequency of obstruction, dislocation, *etc*.
**Microbiology**		
Microbiological history	Clinical records	Review of previous airway cultures and resistances
Current colonisation	Swab/sputum analysis	Current bacterial spectrum (*e.g. P. aeruginosa*)
**Imaging (primary outcome)**		
Structural lung disease	Ultralow-dose PCCT	Bronchiectasis, wall thickening, mucus plugging
Thoracic deformity	Ultralow-dose PCCT	Scoliosis, thoracic cage shape

BMI: body mass index; GI: gastrointestinal; STIKO: Ständige Impfkommission (Standing Committee on Vaccination); CNS: central nervous system; RTI: respiratory tract infection; ICU: intensive care unit; NIV: noninvasive ventilation; IPPB: intermittent positive-pressure breathing; VRP: variable resistance pressure; p-FOIS: Paediatric Functional Oral Intake Scale; DSFS: Drooling Severity and Frequency Scale; PEG: percutaneous endoscopic gastrostomy; PEJ: percutaneous endoscopic jejunostomy; *P. aeruginosa*: *Pseudomonas aeruginosa*; PCCT: photon-counting computed tomography.

#### Speech and swallowing assessment

In addition to item-based documentation, swallowing and drooling are assessed using the Paediatric Functional Oral Intake Scale [38] and the Drooling Severity and Frequency Scale [39], alongside a structured clinical history and examination by an experienced speech and language therapist. This assessment serves to characterise swallowing function and feeding safety and supports the categorisation of participants into relevant clinical subgroups. Furthermore, it provides essential information to identify risk factors for aspiration and secondary pulmonary complications.

#### Chest CT protocol

All chest CT scans are acquired using a PCCT (NAEOTOM Alpha; Siemens Healthineers, Erlangen, Germany), the world's first clinically approved PCCT system. Modified ultralow-dose, noncontrast, paediatric-optimised protocols were implemented for this study. Scans are acquired head-first and supine with arms overhead following inspiratory instructions. The uniform scan parameters are as follows: tube voltage of 100 kVp with tin filtration, detector configuration of 144×0.4 mm and automatic tube current modulation (CAREkV; Siemens Healthineers, Erlangen, Germany), pitch factor of 2.4, and rotation time of 0.25 s. Total chest scan time is <1 s. This ultra-fast technique minimises motion artefacts and eliminates sedation, which is vital for children with neurodisability at high risk of respiratory complications. Standard image quality is level 5. If CT planning indicates a volumetric CT dose index (CTDIvol) >0.2 mGy, the image quality level will be reduced to 4, 3 or 2 to ensure a CTDIvol ≈0.2 mGy. Image datasets will be reconstructed using soft tissue and lung reconstruction kernels (Br40 and Bl60) with an iterative reconstruction strength of 3, a slice thickness of 1.0 mm, and an increment of 1.0 mm in a 1024×1024 matrix. For the *post hoc* calculation of the effective dose, the participants will be categorised into three weight-based groups: 1) 10 to <32 kg (5 to <10 years); 2) 32 to <56 kg (10 to <15 years); and 3) >56 kg (15–18 years).

#### Chest CT analysis

All PCCT scans will undergo independent and blinded review by three board-certified radiologists with extensive experience in thoracic imaging. Readers are blinded to the clinical data. Radiological parameters assessed include segmental and lobar consolidations (including atelectasis and dystelectasis), bronchiectasis, mucus plugging, central airway stenosis, and deformities of the thoracic cage and spine. In uncooperative patients, the respiratory phase will be retrospectively estimated using radiological surrogates, including tracheal wall configuration, posterior rib count, and diaphragmatic position. To avoid misclassifying expiratory artefacts as structural disease, findings will be strictly interpreted within the context of the estimated lung volume.

#### Pulmonary function testing

Pulmonary function tests are performed to assess respiratory capacity and airflow limitation in participants with neurodisability who can participate in standardised testing. Spirometry and peak cough flow (PCF) measurements are conducted according to current paediatric standards [40, 41]. Measurements are performed using calibrated equipment under the supervision of experienced personnel. Parameters recorded include forced vital capacity, forced expiratory volume in 1 s and PCF. Because these tests are unfeasible for many children with severe neurodisability, we additionally systematically record data from recent sleep studies (when available within the preceding 12 months, *e.g.* nocturnal hypoventilation) and detailed parameters of mechanical ventilation (indication and time since initiation) as clinical surrogates for respiratory decline.

### Sample size calculation

Due to the exploratory design and lack of robust prevalence data, the study's primary objective is to estimate and compare the prevalence of structural lung abnormalities on PCCT for the three subgroups (children with tracheostomy, children with neurodisability and dysphagia, and children with neurodisability without dysphagia). Based on retrospective data and clinical experience from our tertiary care centre, we expect structural lung abnormalities to occur in ∼40% of children with tracheostomy, 30% of those with neurodisability and dysphagia, and 10% of those without clinical signs of dysphagia. Sample size calculation using a Chi-squared test assumes differing prevalences between neurodisability groups (alternative hypothesis). Assuming the aforementioned prevalence values and equal group sizes, we calculated, using PASS 2022 (version 22.0.2; NCSS, Kaysville, UT, USA), that a sample size of 147 participants is required for a power of 90% and a significance level of 5%. As all CT examinations follow established clinical pathways at a single centre, we anticipate very few non-evaluable scans, making additional sample size inflation unnecessary. Recruitment will continue until the required number of evaluable CT examinations is obtained. The total recruitment duration is set at 24 months. Therefore, a target of 150 enrolled participants was defined to ensure at least 147 analysable datasets, allowing a 2% dropout rate. To estimate the prevalence for each group, this study aims to include ∼50 patients in each of the three main clinical subgroups. Additional PASS calculations for subgroup sizes of n=50 show the expected precision for different prevalence values. Across plausible prevalence values of 10–50%, the corresponding two-sided 95% confidence intervals have estimated widths of approximately 0.17–0.27 for a group size of 50. For instance, assuming a true prevalence of 10% yields an estimated 95% confidence interval of approximately 4–21%. While these intervals are broad, they remain appropriate for hypothesis generation and for identifying preliminary subgroup-specific patterns in this population. Detailed PASS output for the precision calculations (overall cohort and subgroups) is provided in supplementary tables S1 and S2.

### Statistical analysis

The primary hypothesis assumes that distinct mechanistic pathways drive specific patterns of structural lung abnormalities. Given anticipated baseline imbalances across subgroups, multivariable regression models will adjust for predefined confounders including age, sex, underlying diagnosis, and infection burden, alongside markers of respiratory support to control for disease severity. To account for cohort heterogeneity, the underlying condition will be a categorical covariate, as pathophysiological drivers of respiratory decline (*e.g.* spasticity *versus* muscle weakness) differ between entities. Additionally, an exploratory within-group analysis of the tracheostomy cohort will evaluate differences between proactive (*e.g.* severe NMD) and reactive clinical pathways. Interobserver agreement for imaging assessments will be evaluated using established reliability statistics. Bootstrap resampling will be performed to assess the model stability and robustness of the exploratory findings. Missing data patterns will be analysed descriptively. Given the standardised clinical workflow, we anticipate a low missing rate. However, if the missing data exceed 5% for primary covariates, we will consider multiple imputation methods. Otherwise, a complete case analysis will be performed. Analyses are conducted using R (version ≥4.5.0; R Foundation for Statistical Computing, Vienna, Austria).

### Bias

To minimise selection bias, all eligible patients presenting to the centre are screened consecutively. Information bias is reduced using standardised data collection forms and blinded, multireader radiological evaluations. Residual confounding by disease severity will be addressed by multivariable adjustment in the statistical analyses.

## Discussion

LUCIND is a pioneering, prospective study designed to systematically characterise structural lung disease in children with neurodisability. A major strength is the cross-disciplinary design linking advanced thoracic imaging with clinical, logopaedic and functional data. This prospective approach allows for a systematic correlation of structural abnormalities with infection frequency and airway clearance parameters, providing a multidimensional understanding of disease genesis and early identification markers.

A key innovation of LUCIND is the use of ultralow-dose PCCT. By reducing radiation exposure while maintaining diagnostic image quality [28–30], this technology overcomes ethical barriers to paediatric lung imaging and may set a new standard. Identifying early changes such as bronchial wall thickening, mucus plugging or bronchiectasis even in asymptomatic individuals could shift management from reactive to proactive, aligning with established cystic fibrosis protocols [8, 16].

LUCIND investigates distinct mechanistic endotypes. Tracheotomised children are characterised by dense bacterial colonisation, typically with *Pseudomonas aeruginosa* [12], which provokes a predominantly neutrophilic inflammatory response. In contrast, patients with bulbar dysfunction or oropharyngeal dysphagia frequently exhibit microaspiration and reflux-associated airway injury potentially representing a separate, microaspirative endotype [10, 25]. Differentiating these inflammatory pathways using imaging phenotypes and microbiological patterns could inform targeted therapy. Crucially, this study is going to add important information to multiple national and international recommendations. LUCIND provides the evidence base to operationalise German and British Thoracic Society guidelines regarding structural lung changes and antibiotic stewardship in children with neurodisability [4, 27].

The study benefits from a monocentric design within a high-volume tertiary centre, ensuring standardised imaging and clinical assessments. However, this approach may limit the external generalisability of the initial phase. The broad inclusion and minimal exclusion criteria are intentional, designed to reflect real-world clinical practice and to capture the full spectrum of neurological and respiratory comorbidities, thereby supporting the exploratory character of the study. Future multicentric expansion and integration into national registries would enhance representativeness and enable validation of imaging biomarkers across different healthcare settings.

Potential limitations include recruitment bias towards severely affected patients undergoing advanced respiratory monitoring, and the challenge of performing high-quality CT imaging in uncooperative children. A notable limitation is the inability to achieve standardised inspiratory breath holds in uncooperative patients. Scans acquired during unpredictable respiratory phases may mimic pathology. While retrospective assessment of surrogate markers like tracheal morphology helps estimate the respiratory phase, the lack of standardised inspiration remains an inherent weakness of this vulnerable cohort. Nevertheless, the use of short-breath-hold, noncontrast PCCT protocols eliminates the need for sedation, thus broadening accessibility. Another limitation is the cross-sectional nature of the first LUCIND phase. Longitudinal follow-up will be essential to determine the predictive value of baseline imaging findings for respiratory decline and response to therapy.

In conclusion, LUCIND provides the first descriptive baseline and systematic quantification of secondary structural lung disease in a broad cohort of children with neurodisability. Through the integration of ultralow-dose PCCT and detailed phenotyping, this study aims to establish imaging biomarkers that reflect distinct pathogenic mechanisms. These insights are expected to guide personalised treatment strategies, ranging from antibiotic prophylaxis to aspiration management, and to inform future longitudinal studies and interventional trials. Ultimately, LUCIND aims to shift the focus from the recognition of end-stage bronchiectasis towards the early detection and prevention of secondary lung injury in neurologically impaired children, thereby improving respiratory outcomes and quality of life.

## Data Availability

Data availability is not applicable to this article as no new data were created or analysed in this study design description. Future datasets generated in this study will be available upon reasonable request from the corresponding author.
